# Risk factors associated with persistent coronary artery lesions in children with Kawasaki disease in an Italian cohort

**DOI:** 10.1007/s00431-025-06162-0

**Published:** 2025-05-31

**Authors:** Fiorentina Guida, Elisabetta Morana, Elena Maria Tarì, Leonardo Frazzoni, Laura Andreozzi, Lucia Augusta Baselli, Francesca Lami, Elena Corinaldesi, Cristina Cicero, Lorenzo Mambelli, Gianluca Vergine, Andrea Taddio, Michela Cappella, Barbara Bigucci, Ivana Bruno, Paola Fernicola, Ilaria Frabboni, Matteo Meli, Martina Rossano, Rocco Maurizio Zagari, Marcello Lanari, Marianna Fabi

**Affiliations:** 1 Pediatric Emergency Unit, IRCCS Azienda Ospedaliero Universitaria Di Bologna, Bologna, Italy; 2 Specialty School of Paediatrics (EM, EMT), Alma Mater Studiorum, University of Bologna, Bologna, Italy; 3 UOC Gastroenterologia ed Endoscopia Digestiva Forlì-Cesena, AUSL della Romagna, Forlì-Cesena, Italy; 4 Department of Medical and Surgical Sciences, University of Bologna, Bologna, Italy; 5 Pediatric Intermediate Care Unit, Fondazione IRCCS Ca’ Granda Ospedale Maggiore Policlinico, Via Della Commenda 9, 20122 Milan, Italy; 6 Department of Medical and Surgical Sciences for Mothers, Children and Adults, University of Modena and Reggio Emilia, Modena, Italy; 7 Paediatric Unit, Carpi Hospital, 41012 Carpi, Italy; 8 Department of Pediatrics, AUSL, Guglielmo da Saliceto Hospital, Piacenza, Italy; 9 Department of Paediatrics, Santa Maria Delle Croci Hospital, AUSL Della Romagna, Ravenna, Italy; 10 Pediatric Clinic, Rimini Hospital, AUSL Romagna, Rimini, 47921 Italy; 11 Institute for Maternal and Child Health, IRCCS “Burlo Garofolo”, and University of Trieste, Trieste, Italy; 12 AUSL Bologna, Ospedale Maggiore, Bologna, Italy; 13 Paediatrics Unit, Santa Maria Nuova Hospital, Azienda Unità Sanitaria Locale (AUSL)-Scientific Institute for Research and Healthcare (IRCCS) of Reggio Emilia, 42123 Reggio Emilia, Italy; 14 Paediatrics Unit, G.B. Morgagni Pierantoni Hospital, Azienda Unità Sanitaria Locale (AUSL) Romagna, 47121 Forlì, Italy

**Keywords:** Kawasaki disease, Persistent coronary artery lesions, Risk score, Coronary artery aneurysms, Risk factors

## Abstract

Kawasaki disease (KD) can be complicated—particularly during the acute phase—by coronary artery lesions (CALs). The persistence of CALs (pCALs) beyond the subacute phase increases the risk of long-term cardiovascular morbidity and life-threatening events. While several risk scores, primarily based on Asian and American populations, have been proposed to predict CALs or treatment resistance, few studies have focused on identifying risk factors for pCALs. This study aimed to identify risk factors for pCALs in Italian patients and to evaluate the validity of an existing risk score developed in a North American cohort. Data from KD patients across 11 Italian centers were collected in a centralized RedCap database. pCALs were defined as CALs persisting 8 weeks post-diagnosis. Clinical, demographic, and laboratory features of patients with and without pCALs were compared. Independent risk factors were identified using multiple logistic regression. The predictive performance of Son’s risk score was assessed through ROC analysis. A total of 517 children (87.4% Caucasian) were enrolled; 52 developed pCALs. pCALs were more common in males (12.03%, *p* = 0.06), patients < 6 months (61.5%, *p* = 0.05), those with Asian ethnicity (26.9%, *p* = 0.026), incomplete clinical presentation (*p* = 0.03), and in those with abnormal abdominal ultrasound findings (*p* = 0.04). Affected children had higher WBC, elevated CRP (> 13 mg/dL), and lower hemoglobin. Compared to those with acute CALs, patients with pCALs were younger, more often IVIG non-responders (34.6% vs. 29.6%, *p* < 0.001), and late-treated. Son’s score showed good predictive ability for pCALs.

*Conclusions*: Male sex, younger age, incomplete presentation, Asian ethnicity, and elevated CRP are independent risk factors for pCALs in Italian children with KD. Son’s score may help identify high-risk patients who could benefit from closer follow-up and early adjunctive therapy, even in predominantly Caucasian populations.

**Table:** 

**What is Known:** • *Kawasaki disease can cause CALs, which increase cardiovascular risk if they persist* • *Previous research has focused mainly on predicting CALs or treatment resistance, but little evidence is available on the risk factors for CALs persistence* **What is New:** • *Our study identifies independent risk factors for pCALs in Italian children: male gender, younger age, incomplete presentation, Asian ethnicity, and high CRP levels* • *By applying the Son Risk Score to our population, we confirmed its predictive value in a predominantly Caucasian cohort and its reliability in identifying susceptibility to CALs persistence*

**What is Known:**

• *Kawasaki disease can cause CALs, which increase cardiovascular risk if they persist*

• *Previous research has focused mainly on predicting CALs or treatment resistance, but little evidence is available on the risk factors for CALs persistence*

**What is New:**

• *Our study identifies independent risk factors for pCALs in Italian children: male gender, younger age, incomplete presentation, Asian ethnicity, and high CRP levels*

• *By applying the Son Risk Score to our population, we confirmed its predictive value in a predominantly Caucasian cohort and its reliability in identifying susceptibility to CALs persistence*

## Introduction

Kawasaki disease (KD) is an acute febrile systemic vasculitis with an unclear etiology that primarily affects children under 5 years old [1, 2]. Its major complication is coronary involvement, where vasculopathic processes lead to vascular wall injury and subsequently dilation or aneurysms [3], increasing cardiovascular risk of the affected children in the short, medium [4], and long term [5]. The genetic background [6] affects the susceptibility to the disease [7, 8], and its severity in Asian and other ethnic groups [9–15]. Other predisposing factors, such as environmental factors and infections, interplay with genetics, triggering the disease and favoring the development of coronary artery lesions (CALs) [16–18]. Identifying anamnestic, demographic, clinical, and laboratory variables associated with the risk of coronary involvement could lead to identifying children who might benefit from additional immunomodulatory therapy alongside intravenous immunoglobulins (IVIG) at an early stage, thereby reducing vascular damage and improving cardiovascular outcomes. Indeed, CALs develop in 5% of patients despite proper and timely treatment making KD the leading cause of acquired heart disease in children [19]. CALs typically develop within the first ten days after the onset of KD [20] and tend to normalize their diameter in up to 75% of cases within 2 years of the disease’s diagnosis [20, 21] despite vascular wall histological alteration occurring.

So far, numerous studies have been conducted to identify risk factors that expose children with KD to developing CALs and their persistence over the first 8 weeks of the diagnosis. Younger age, male gender, IVIG resistance or a delayed administration, a prolonged course of fever, multiple coronary involvement, along with blood test alterations, such as elevated C-reactive protein (CRP), altered neutrophil-to-lymphocyte ratio, reduced hemoglobin level, and hypoalbuminemia, have been related to higher rate of CALs in the acute stage of KD [22–27]. In addition, subgroups of children with KD with different laboratory and clinical presentations have been associated with coronary involvement or IVIG resistance [28, 29].

Moreover, different risk scores aimed at identifying the formation of CALs have been validated in the Asian population [30–32] but showed lower accuracy in other ethnicities [33, 34]. The risk score published by Son et al. assessed the predictive value for coronary lesions in a North American multiethnic cohort using simple data, such as age, ethnicity, CRP, and initial coronary z-score [35]. It is thus necessary to identify risk factors associated with a higher likelihood of persisting after the subacute stage and to validate existing diagnostic tools across different geographical regions. This will enable patients to benefit from personalized treatments based on precise risk stratification.

## Materials and methods

Data for this study were retrospectively and prospectively collected and entered into a REDcap database, from an Italian cohort composed of 11 recruiting centers [36] between January 1, 2000, and June 30, 2023.

This study was performed in line with the principles of the Declaration of Helsinki. Approval was granted by the Ethics Committee of IRCCS AOU BO (Avec 340/2017/O/OssAOUBO approved on 1/16/2019). According to local regulations, the institutional review board granted the study approval at each enrolling site. Informed consent was obtained from all individual participants’ parents included in the study.

The Data Coordinating Center (IRCCS Azienda Ospedaliero-Universitaria di Bologna, Italy) reviewed and analyzed all the data, ensuring patient eligibility, data completeness, and accuracy.

The materials and methods have been previously detailed in a prior publication by our group [36]. Briefly, the diagnosis of KD, the clinical presentation (complete and incomplete form), the response to treatment with IVIG (IVIG responders, IVIG nonresponders), and the timing of treatment related to the diagnosis (late or not treated) were based on the American Heart Association criteria [1, 4].

Collected data included demographical and clinical features, blood tests (i.e., white blood cells (WBC), percentage of neutrophils (N%), percentage of lymphocytes (L%), hemoglobin levels (Hb), platelet count (PLT), serum glucose, hepatic and kidney function markers, CRP and erythrocyte sedimentation rate (ESR), serum albumin, and serum electrolyte levels), echocardiographic evaluation, and abdominal ultrasound (US) performed during the hospitalization if requested by the physician. The laboratory values were collected during the acute (from the onset to the 10 th days after fever onset), subacute (from the 11 th to the 20 th days after fever onset), and chronic stage (from the 6 th week after fever onset) for each patient.

In addition, gastrointestinal (GI) involvement was defined as the presence of symptoms such as diarrhea, abdominal pain, or vomiting, along with abnormal abdominal US findings, including gallbladder hydrops, effusion in the pouch of Douglas, thickening of the intestinal wall, mesenteric adenopathies, or abdominal effusion. Echocardiography was performed during the acute, subacute, and chronic stages with the same modalities previously published [36]. CA involvement was then classified by Z-score as no involvement (Z < 2), dilation (Z 2 to < 2.5), small aneurysm (Z 2.5 to < 5), medium aneurysm (Z 5 to < 10), and large aneurysm (Z > 10), according to the 2017 AHA guidelines [1]. Persistent coronary artery lesions (pCALs) were defined as the persistence of CA dilation or aneurysm 8 weeks after the diagnosis of KD. Transient coronary lesions (tCALs) were defined as CALs development by the 20 th day of fever and resolved by 8 weeks after fever onset. Left main coronary artery (LMCA), left anterior descending coronary artery (LAD), circumflex (CX), and right coronary artery (RCA) were studied.

Categorical variables were presented as absolute frequency and percentage, while non-normally distributed continuous variables were displayed as mean, standard deviation (SD), and/or interquartile range (IQR). Multivariate logistic regression was used to assess the risk factors for developing persistent coronary artery lesions (pCALs) in our population. Odds ratio and 95% confidence interval were obtained, and *p* < 0.05 was considered as statistically significant. The variables statistically related to a higher risk for developing pCALs were evaluated to elaborate a risk prediction model. A receiver operating characteristic (ROC) analysis was conducted to assess the performance and reliability of Son’s risk prediction model [35] to predict the development of pCALs in the presented population. Statistical analysis was performed using STATA software version 16 (Stata Corp., College Station, TX).

## Results

A total of 517 (boys 53.8%, mean age 44 months, SD 38.19) patients were included in the study and 52 patients (10%) developed pCALs.

Demographical, clinical, therapeutical, laboratory, and radiological features of patients with and without pCALs are displayed in Table 1.

**Table 1:** Demographical clinical and laboratoristic features of patients with pCALs compared to those without pCALs

	pCALs		No pCALs		*p* value	OR	IC (95%)
	*N*	%	*N*	%			
Demographical features							
Gender Male Female	38 14	74.5 27.5	278 187	53.8 36.2	0.043*		
Ethnicity Asian Caucasian Afro-American Other	7 40 3 2	13.7 78.4 5.9 3.9	19 412 15 19	3.7 79.7 2.9 3.7	0.019*		
Seasonality Autumn Summer Winter Spring	13 10 16 13	25.5 19.6 31.4 25.5	118 66 158 120	22.8 12.8 30.6 23.2	0.792		
Clinical features							
Clinical presentation Complete Incomplete	25 26	49 51	295 164	57.1 31.7	0.033*		
Fever duration, mean days (SD)	13	(7.6)	8.6	(4.1)	< 0.001*		
Persistent fever (> 10 days)	48	94.1	428	82.8	0.015*		
Conjunctivitis	30	58.8	213	41.2	0.103		
Swelling of the hand and feet	20	39.2	148	28.6	0.333		
Rash	32	62.7	221	42.7	0.155		
Oral changes^a^	26	51	208	40.2	0.469		
Cervical adenopathy^b^	15	29.4	154	29.8	0.533		
GI group^c^	29	55.8	266	57.3	0.843		
GI manifestations^d^	16	30.7	179	34.6	0.276		
Liver involvement^e^	0		10	1.9			
Abdominal ultrasound findings^f^	5	9.6	38	7.4	0.040*		
Pulmonary complications^g^	2	3.9	20	3.9	0.561		
Perianal/diaper erythema	3	5.9	9	1.7	0.082		
Osteomuscular involvement^h^	0		10	1.9	0.286		
Aseptic meningitis	0		1	0.2	0.738		
Facial nerve palsy	0		1	0.2	0.738		
Macrophagic activation syndrome	0		1	0.2	0.738		
Retropharyngeal edema/phlegmon	0		1	0.2	0.738		
Treatment							
Standard treatment	46	88.5	444	96.7	0.023*	OR 1.08	95% CI 0.46–2.48
Late treatment	12	23	51	11.1	0.076	OR 2.67	95% CI 1.32–5.41
Not treated	6	11	15	3.3	0.023*	OR 4.27	95% CI 1.58–11.53
IVIG responders	22	42.3	342	83.8	< 0.001*	OR 0.36	95% CI 0.20–0.64
IVIG nonresponders	18	34.6	102	23	< 0.001*	OR 2.09	95% CI 1.13–3.84

*GOT* glutamic oxaloacetic transaminase, *GPT* glutamate-pyruvate transaminase, *GGT* gamma-glutamyltransferase, *ESR* erythrocyte sedimentation rate, *CRP* C-reactive protein

*Statistical significance

^a^Oral changes: erythema and cracking of lips (cheilitis); strawberry tongue; erythema of oral and pharyngeal mucosa

^b^Cervical adenopathy: defined as a swallowing of the cervical nodes bigger than 1.5 cm

^c^GI, gastro-intestinal manifestations: diarrhea and/or abdominal pain and/or vomiting

^d^GI, gastro-intestinal group refers to any patient with one of the following: liver test anomalies (GOT and GPT), pathological findings at abdominal ultrasounds, GI signs or symptoms

^e^Liver involvement: liver test anomalies and/or jaundice

^f^Abdominal ultrasound findings: gallbladder hydrops, effusion in the pouch of Douglas, swallowing of the intestinal wall, mesenterial adenopathies. abdominal effusion

^g^Pulmonary complications: peribronchial interstitial inflammatio

^h^Osteomuscular involvement: joint pain and/or arthritis

Compared to patients without pCALs, those with pCALs were younger (mean age 25.3 months, SD 24.8; *p* 0.043) and predominantly male (38/52, 74.5%; *p* = 0.043), more likely to be Asian (7/52, 13.7%; *p* = 0.019), to have an incomplete clinical presentation (26/52, 51% vs 164/465, 31.7%, *p* 0.033) and fever lasting more than 10 days (48/52, 94.1% vs 428, 82.8%, *p* 0.001). They were more prone to present abdominal US anomalies (respectively 5/52, 9.6% vs 38/517, 7.4%, *p* 0.04), with a similar rate of GI manifestations when compared to children without pCALs (respectively 16/52, 31.4% vs 179/517, 34.6% *p* 0.276).

Forty-six out of 52 (88%) who developed pCALs received IVIG, and 34/46 (73.9%) within the first 10 days of fever. Twenty-two out of 46 (47.8%) patients were IVIG responders, 18 patients (39.1%) were IVIG non-responders. Second line treatments were a second dose of IVIG infusion alone in 6/46 (13.05%), IVIG and steroids in 7/46 (15.2%), and biologics in 5/46 (10.9%), particularly anakinra in 4 (8.7%) patients and infliximab in 1 (2.2%). The lack of treatment with IVIG and IVIG unresponsiveness were significantly associated with pCALs (respectively *p* 0.023; OR 4.27, 95% CI 1.58–11.53, and *p* < 0.001; OR 2.09, 95% CI 1.13–3.84). Table 2 shows laboratory tests of the patients with pCALs compared with tCALs, and without pCALs.

**Table 2:** Comparison of laboratoristic data of patients with pCALs with tCALs (*p* value^a^) and with those who never developed CALs (*p* value^b^)

	tCALs	*p* value^a^	No pCALs	*p* value^b^	pCALs
Red blood cells × 10^12^ (/L), mean ± SD	4.239 ± 1.115	0.087	4.327 ± 1.104	0.030*	4.145 ± 1.676
Hemoglobin (g/dL), mean ± SD	10.9 ± 1.43	0.042*	11.15 ± 1.3	0.079	10.7 ± 1.2
Platelets (× 10^9^/L), mean ± SD	376.577 ± 177.540	0.844	372.397 ± 180.737	0.605	386.956 ± 187.281
GOT (UI/L), mean ± SD	62.116 ± 48.4	0.481	71.6 ± 40.4	0.726	62.3 ± 31.9
GPT (UI/L), mean ± SD	68.7 ± 48.83	0.323	77.7 ± 45.4	0.647	87.1 ± 62.8
GGT (UI/L), mean ± SD	52.148 ± 32.74	0.801	52.2 ± 33.67	0.441	70.2 ± 57.3
Albumin (g/dL), mean ± SD	3.475 ± 0.67	0.008*	3.4 ± 0.68	0.003*	3.1 ± 0.667
Sodium (mmol/L), mean ± SD	134.47 ± 3.2	0.837	134.5 ± 3.78	0.746	134.3 ± 2.7
ESR (mm/h), mean ± SD	62.08 ± 31.12	0.701	63.8 ± 32.7	0.630	60.2 ± 33.1
CRP (mg/dL), mean ± SD	11.01 ± 7.69	0.008*	9.3 ± 6.5	0.001*	12.7 ± 7.7

Asterisks indicate statistical significance

 When compared with patients without pCALs, those with pCALs presented lower red blood cell count (*p* 0.030) and serum albumin (*p* 0.003) and higher CRP (*p* 0.001), and lower Hb and albumin (respectively *p* 0.042 and *p* 0.008) and higher CRP (*p* 0.008) when compared with patients with tCALs.

In addition, in comparison to patients with tCALs (35), those with pCALs were younger (mean age 25.3 months versus 36.5 months; *p* 0.043), IVIG resistants (34.6%, 18/52 versus 29.6%, 153/517; *p* < 0.001); late treatment was more frequent in pCALs without reaching the statistical significance (11.5%, 6/52 versus 7.9% 41/135; *p* 0.076). Both groups experienced a similar incidence of GI involvement (16/52, 31.4% vs 84/135, 26%; *p* 0.868).

pCALs were classified as dilation in 48.0% (25/52), small aneurysm in 26.9% (14/52), medium aneurysm in 17.3% (9/52), and giant aneurysm in 7.7% (4/52) of the population. When pCALs occurred in a single vessel, LAD was the most frequently affected (20/52, 38.4%), followed by the Cx (15/52, 28.8%), LMCA (13/52, 25%), and RCA (4/52, 7.7%). Multi-coronary injury (at least 2 vessels involved) occurred in 41/52 (78.8%) of patients: LAD was affected in 59.6% (31/52), LMCA in 51.9% (27/52), RCA in 40.4% (21/52), and Cx in 36.5% (19/52). All patients presented normal left ventricular systolic function, 7.6% (4/52) mild mitral regurgitation, and 3.8% (2/52) mild pericardial effusion.

tCALs developed in 129/135 (95.5%) patients during the acute phase and 6/135 (4.4%) during the convalescent phase. CALs persisted in 52/135 (38.5%) and regressed in 83/135 (61.5%).

The total number of CALs during acute and subacute stage was 111: LMCA was the most commonly affected vessels (44/111; 39.6%), followed by LAD (23/111; 20.7%), Cx (25/111; 22.5%), and finally proximal RCA (19/111; 17.1%). At 8 weeks after the diagnosis (Table 3), the regression rate followed the initial severity: 62.2% if the initial lesion was dilation, 32.4% if small aneurysms, 5.4% if medium aneurysms and none of initial large aneurysms.

**Table 3:** Eight-week regression rates of CALs developing during the acute and subacute stage

	Regression rate of tCALs based on initial severity and site				
	LMCA	LAD	Cx	RCA prox	Total
Dilation	27 (61.4%)	14 (60.9%)	14 (56%)	14 (73.7%)	69 (62.2%)
Small aneurysms	15 (34.1%)	8 (34.8%)	9 (36%)	4 (21.1%)	36 (32.4%)
Medium aneurysms	2 (4.5%)	1 (4.3%)	2 (8%)	1 (5.3%)	6 (5.4%)
Large aneurysms	0	0	0	0	0
Total	44 (39.6%)	23 (20.7%)	25 (22.5%)	19 (17.1%)	111

A multivariate logistic regression was conducted to assess risk factors for developing pCALs. Male gender (*p* 0.043, OR 2.34, IC95% 1.02–5.34), age younger than 6 months (*p* 0.042, OR 0.37, IC95% 0.14–0.96), Asian ethnicity (*p* 0.019, OR 0.63, IC95% 0.44-0.93), fever lasting more than 10 days (*p* 0.015, OR1.56, IC95% 1.09–2.25), CRP higher than 13 mg/dL (*p* 0.033, OR 2.19, IC95% 1.06–4.52) were independent risk factors for pCALs, while Hb lower than 10.3 g/dL (*p* 0.079, OR 1.97, IC95% 0.92–4.2) was not statistically associated with higher incidence of pCALs. The cutoff of 10.3 g/dL was chosen as the lowest standard deviation for the definition of anemia in children aged from one to 12 years old (37).

**Fig. 1:**
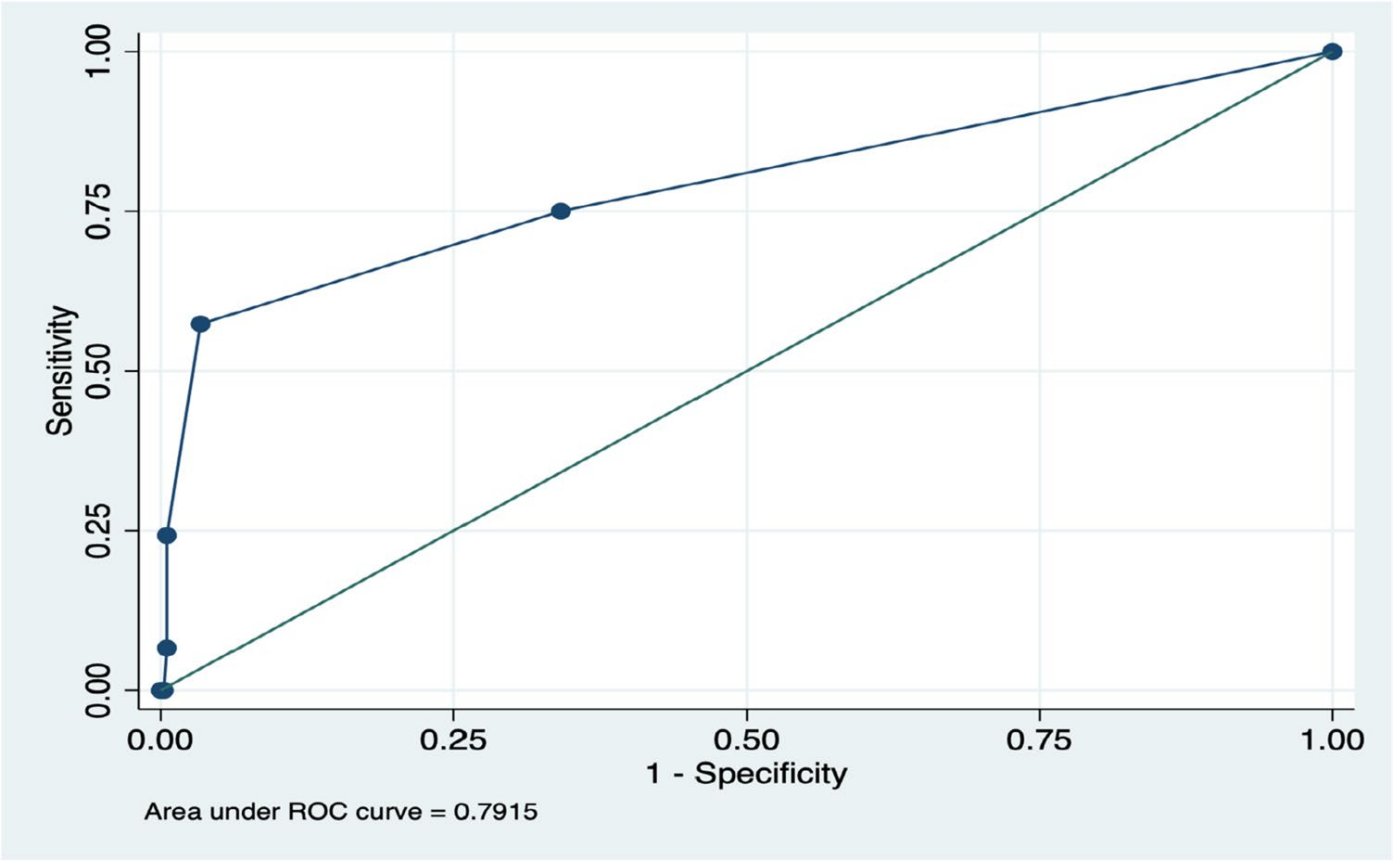
The application of Son’s risk score to predict the development of pCALs in the presented population. The ROC curve evaluates the performance of Son’s risk prediction model (35) in predicting the development of pCALs. The *X*-axis represents the false positive rate (1—Specificity), and the *Y*-axis represents the true positive rate (Sensitivity). The blue curve illustrates the model’s ability to distinguish between positive and negative classes at various threshold settings. The green diagonal line signifies random classification. The area under the ROC curve (AUC) is 0.7915, indicating that the model has good discriminative power

A ROC analysis was performed to assess the performance of Son’s risk prediction model (35) to identify patients at risk for developing pCALs in our cohort. The area under the ROC curve (AUC) is 0.7915, suggesting the good discriminating power of the model (Fig. 1).

## Discussion

Our study confirms that male gender, younger age, Asian ethnicity, incomplete clinical presentation, longer fever duration (particularly more than 10 days), abnormal abdominal US findings, and IVIG resistance are risk factors for the persistence of coronary lesions in an Italian mostly Caucasian cohort. At the laboratory tests, lower red blood cells, lower albumin, and higher CRP are associated with persistence of coronary damage. By the comparison of patients with pCALS and tCALs, the former were younger and more likely to be IVIG resistant and late treated, to have lower hemoglobin and albumin and higher CRP.

Thus, younger age, no response to standard treatment, higher inflammatory marker and lower albumin are linked in general to coronary involvement either during acute and 8-weeks phases of KD, potentially supporting the role of a longer inflammation in younger subjects.

In addition, IVIG resistance was an independent risk factor, as age younger than 6 months, Asian ethnicity, fever duration more than 10 days and CRP greater than 13 mg/dL, according with data from multiethnic children with KD.

Most studies tried to identify risk factors for CALs during the acute stage of KD [26, 36, 37], and fewer focused on the factors linked to the coronary damage over time [22]. Our findings show that IVIG resistance is linked with the occurrence of coronary damage not only during the acute stage of the disease [1, 36], but also with its persistence after resolution of inflammation. This is crucial because IVIG resistance, associated with large CALs and male gender, was significantly linked with major cardiac adverse events in a multicentric Japanese study including over 1000 KD patients [38].

In our cohort, CALs persisted for 8 weeks after diagnosis in 38.2% of patients after the acute phase [36]. Coronary size is known to influence lesion progression [39, 40]: small aneurysms typically regress, while 70–95% of medium- and large-sized aneurysms tend to persist, with up to 20% progressing to stenosis. In addition, smaller CALs tend to regress earlier [41], usually within six months from the onset, especially in patients younger than one year of age.

Our results show that smaller CALs, including dilations and small-sized aneurysms, regress after 8 weeks in over 90% of cases, whereas large and giant coronary aneurysms do not. The lack of improvement of coronary size in case of large/giant aneurysms is different from what previously found in KD Japanese patients, who showed a regression rate of 28–36% of cases 10 years after the diagnosis [20]. It is important to highlight the different time points, as our data confirm the trend of size-based improvement and reflect an evolving condition, given that pathological mechanisms continue for years after diagnosis [3].

The most common sites of coronary involvement in decreasing frequency are reported to be the proximal LAD, proximal RCA, LMCA, and CX [20, 42]. In our population, the distribution of pCALs is consistent with data from multiethnic and Asian population: LAD was the most affected coronary artery in case of single and multivessel involvement, followed by CX and LMCA; RCA, on the other hand, was more frequently involved when multicoronary injury occurred. The vast majority of CALs were dilation and small aneurysms, accounting for 74.8% of cases, while giant aneurysms persisted in 7.6% of patients.

Blood tests linked to coronary damage mostly overlap for patients with aCALs and pCALs: lower values of red blood cells, Hb and albumin, and higher CRP levels were significantly associated with tCALs and pCALs, in line with other cohorts [36, 43, 44]. Moreover, a higher hemoglobin level was identified as an independent risk factor for CAL regression within three weeks of diagnosis [45] and is therefore included in a nomogram score to predict it.

Our findings indicate that significant and persistent inflammation—characterized by elevated CRP levels, lower albumin, prolonged fever duration, and IVIG resistance—plays a crucial role in coronary damage. Previous studies have demonstrated an association between CRP [45, 46] and IVIG unresponsiveness [29, 47] with the development of CALs. Notably, CRP emerged as an independent risk factor for the persistence of CALs in our cohort. Furthermore, CRP levels > 13 mg/dL, which are included in the Son’s score, demonstrated a strong predictive value for injury persistence in our study population.

Despite its link to IVIG resistance, pCALs were detected in over 40% of IVIG responders. Additionally, Asian ethnicity was identified as an independent risk factor, even though only 13.7% of the cohort was of Asian descent. This finding supports the hypothesis that coronary injury arises from a complex interplay of multiple factors, including genetic predisposition and unknown environmental triggers.

The presence of abdominal US anomalies during the acute phase of the disease was significantly associated with pCALs. Notably, previous studies linked abdominal US anomalies to a more severe course of KD, including the development of CALs [29] and IVIG resistance [48]. Additionally, elevated fecal calprotectin levels and aCALs have been shown to predict CAL persistence [48].

However, in our population, the gastrointestinal (GI) group—including those with GI symptoms and/or liver laboratory abnormalities—did not exhibit a higher risk for pCALs. This finding aligns with data from a multiethnic population [28] but contrasts with a Chinese cohort that demonstrated an intermediate risk for CALs [48].

Similarly to our findings, both studies identified younger age at diagnosis as the highest risk factor for CALs compared to groups with liver involvement, severe inflammation, cervical lymphadenopathy, and elevated band neutrophils. Furthermore, gamma-glutamyl transferase levels were reported as an independent risk factor for CAL persistence in another Asian cohort [22]; however, we did not observe this association in our study.

KD cardiovascular *sequelae* are related to coronary damage leading to stenosis and thrombosis, myocardial ischemia, and sudden death [19, 49]. Multiple studies across different ethnic groups [21, 22, 35, 49–52] have emphasized the significance of initial coronary size, which appears to be correlated with CAL persistence. Therefore, it is essential to develop and validate a predictive tool for pCALs in KD patients. Son et al. developed and validated a risk score model in a multiethnic US population, including initial coronary artery size and simple clinical and laboratory data, such as age, ethnicity and CRP [35]. The Update on Diagnosis and Management of KD stated that patients identified as “high-risk” by Son risk score could benefit from initial intensification treatment [2]. When tested in our population, Son’s Risk Score demonstrated strong predictive value for pCALs, suggesting its effectiveness in assessing the persistence of CALs in patients who could benefit from the intensification treatment. For instance, primary adjunctive treatment with Infliximab [53] or Anakinra [20] were shown the be associated with a greater likelihood of CALs regression in patients with coronary involvement at the initial evaluation.

## Conclusion

In a predominantly Caucasian cohort, IVIG resistance, diagnosis of KD before 6 months of age, Asian ethnicity, and CRP levels exceeding 13 mg/dL were identified as independent risk factors for CAL persistence beyond 8 weeks post-diagnosis. Abnormal abdominal ultrasound findings were associated with pCALs and should be assessed at diagnosis, particularly in boys with an incomplete clinical presentation, anemia, and low albumin levels. Notably, Son’s score enables the rapid identification of patients at risk for persistent coronary injury, allowing for early treatment intensification regardless of IVIG response, potentially reducing KD-related morbidity.

## Data Availability

Data for this study were retrospectively and prospectively collected and entered into a REDcap database, from an Italian cohort composed of 11 recruiting centers between January 1, 2000, and June 30, 2023.

## Abbreviations

AUC: Area under the curve
CA: Coronary artery
CAL: Coronary artery lesion
CRP: C-reactive protein
Cx: Left circumflex branch
ESR: Erythrocyte sedimentation rate
GGT: Gamma-glutamyltransferase
GOT: Glutamic oxaloacetic transaminase
GPT: Glutamate-pyruvate transaminase
IQR: Interquartile range
KD: Kawasaki disease
LAD: Left anterior descending coronary artery
LMCA: Left main coronary artery
OR: Odds ratio
pCALs: Persistent coronary artery lesions
RCA: Right coronary artery
ROC: Receiver operating characteristic
SD: Standard deviation
